# Analysis and preliminary validation of the molecular mechanism of fat deposition in fatty and lean pigs by high-throughput sequencing

**DOI:** 10.1007/s00335-019-09795-3

**Published:** 2019-03-06

**Authors:** Jing Xiang Cui, Qi Fan Zeng, Wei Chen, Hong Zhang, Yong Qing Zeng

**Affiliations:** 1 College of Animal Science and Technology, Shandong Agricultural University, Taian, 271018 China; 2 Weifang University of Science and Technology, Shouguang, 262700 Shandong China; 3 Ministry of Education Key Laboratory of Marine Genetics and Breeding, College of Marine Science, Ocean University of China, Qingdao, 266003 China; 4 Department of Endocrinology, Huai’an First People’s Hospital, Nanjing Medical University, Huai’an, 223300 Jiangsu China

## Abstract

Fat deposition in muscle includes intramuscular fat (IMF) and intermuscular fat. IMF content is an index of pork quality; however, because IMF content is difficult to measure in vivo in young animals, conventional breeding for IMF content is difficult to carry out. The mechanism and progression of animal fat deposition is not well understood, and there are currently no effective control methods. In this study, using Laiwu and large white pigs as the research subjects and RNA sequencing technology, we analyzed the genetic mechanism of animal fat deposition in pigs. Specifically, we analyzed the features of lncRNAs and their potential target genes. We obtained 464 million clean reads, from which 907 lncRNAs were identified. The *cis* and *trans* analysis identified target genes, including genes that were upregulated (286) and downregulated (621) in the fatty and lean pigs. ENSSSCG00000008692_ADD1, ENSSSCG00000023124_ADD1 and ENSSSCG00000005918_DGAT1 were validated as target genes of the lncRNAs and were shown to be closely related to fat deposition. These results provide a basis for studying the different metabolic lncRNA expression of IMF deposition. In addition, as the valuable model animal to study the mechanisms of obesity, pigs may represent a new avenue for studying human obesity.

## Introduction

Animal fat deposition is a complex biological process. If fat accumulation is abnormal or excessive, it will lead to obesity, which is harmful for animal and human health (Wei et al. 2016a, b). Fat deposition in muscle includes intramuscular fat (IMF) and intermuscular fat. IMF refers to muscle connective tissue membranes (epimysium) contained within the muscle and is considered an important meat quality trait.

The IMF content is an index of the pork quality and has a significant positive effect on meat tenderness and flavor (Hausman et al. 2009). Because the IMF content is difficult to measure in vivo in young animals, conventional breeding to increase IMF content is difficult to carry out. Improving the IMF content while ensuring the lean meat rate has become a new target for breeders. However, the mechanism and progression of animal fat deposition are not well understood, and there are currently no effective control methods.

Recent investigations of adipose development suggest that a significant number of long noncoding RNAs (lncRNAs) participate in the regulatory networks of adipogenesis and play a key role in regulating adipogenic commitment and differentiation (Xu et al. 2010; Sun et al. 2013). LncRNAs are an emerging class of regulators involved in a myriad of biological processes. Obesity is a significant risk factor for several serious human diseases, such as type 2 diabetes, cardiovascular disease and certain types of cancers (Després and Lemieux 2006; Dodson et al. 2013). Therefore, fully revealing the molecular regulation mechanisms of fat deposition involves determining both the genes related to fat deposition and the genes that are differentially expressed between animals with high and low fat deposition.

Laiwu pigs (here after LW) are known for their high fat deposition and thus represent the good animal model for studying the mechanisms of high intramuscular fat deposition (Spurlock and Gabler 2008; Chen et al. 2013). In contrast, Large White pigs (hereafter DB) are a lean-type pig breed characterized by a high growth rate and body weight (Grzes et al. 2016). Therefore, in this study, we used both fatty- and lean-type pigs to perform high-throughput sequencing to determine the differential expression of lncRNAs and mRNAs between the two breeds. The aim of this study was to elucidate the lncRNA differentiate expression in these two kinds of pigs for a better understanding of its molecular genetic controls. Determining the genes related to fat deposition and validating their functions is a top priority for the treatment of human obesity and related diseases. Further understanding the molecular mechanisms that control adipogenesis is critical for improving pork meat quality and identifying new targets for combating obesity.

## Results

### The characteristics of pigs

The IMF content and backfat thickness (BFT) were determined in 60 pigs. The average IMF content of LW (12.78 ± 1.02%) was significantly higher than that of DB (1.15 ± 0.07%). The results indicated that the IMF content was significantly different between the fatty- and lean-type breeds (P < 0.01). With regard to the BFT, LW was significantly thicker than DB (P < 0.01) (Cui et al. 2015). Three LW and three DB pigs were used for the deep sequencing analysis to determine how lncRNAs and mRNAs affect IMF development. Several important lncRNAs and mRNAs previously shown to regulate target genes that are closely related pig fat deposition were analyzed in this study.

### Reads and mapping results of the RNA deep sequencing

**Fig. 1:**
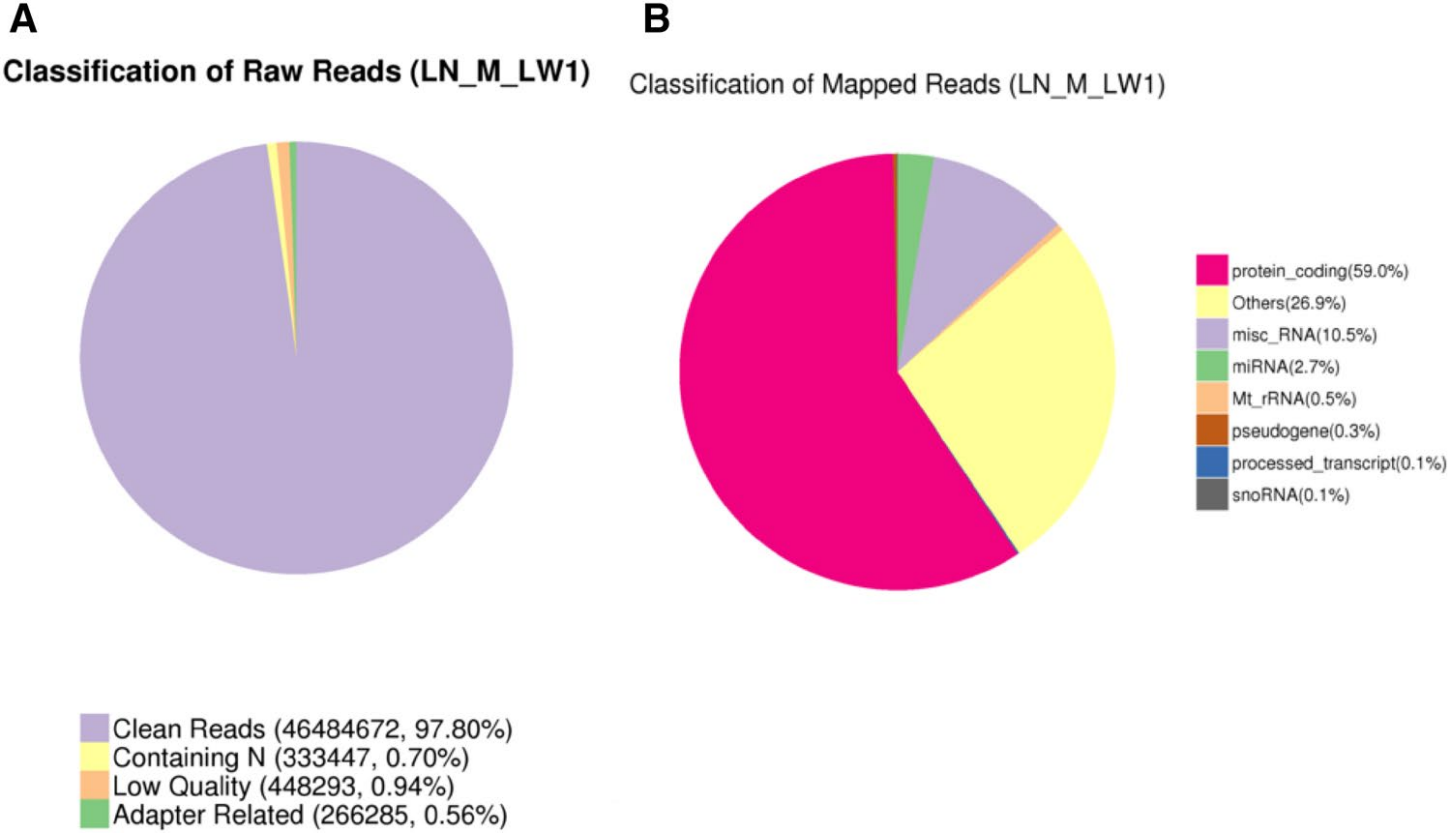
Reads and mapping characteristics of the lncRNAs. **a** The LN_M_LW1 pig clean reads. The number of reads was counted from single-end sequencing. **b** Reads and mapping results for the RNA deep sequencing. The percentage of mRNA reads was approximately 60%, followed by tRNAs, miscRNAs and others for the LN_M_LW1 pig

The two libraries (LW and DB) were collected from the longissimus muscle tissue and contained totals of 302 and 307 million raw reads (obtained from paired-end sequencing) in the range of 16–32 nt, with error rates of less than 0.01% and 0.02%, respectively. After filtering the raw reads and removing the low-quality reads, there were approximately 295 and 300 million clean reads (LW and DB, respectively). As an example, the Pig LN_M_LW1 library had 97.80% clean reads (Fig. 1a, obtained from single-end sequencing). The clean reads were mapped to the pig genome, and the total mapped reads constituted more than 70% of the clean reads. Uniquely mapped reads constituted greater than 60% of the total, while the nonspliced reads were more than 43%, and the spliced reads were approximately 19.55%. The majority of the reads corresponded to protein-coding RNAs (59%), followed by microRNAs, tRNAs and others (Fig. 1b). The sequencing analyses were performed for the fatty and lean groups by the Novogene Bioinformatics Technology Company.

### The RNA-seq data assessment

Cuffdiff (v2.1.1) was used to calculate the fragments per kilo base of exon per million fragments mapped (FPKMs) of both the lncRNAs and coding genes in each sample (Trapnell et al. 2010). The FPKM is a parameter calculated based on the length of the fragments and read counts mapped to each fragment. The expected FPKM values were used to calculate the gene expression levels of the fatty and lean groups. The RNA-seq Pearson correlation coefficients of the gene expression levels were greater than 0.85 in the fatty group and greater than 0.84 in the lean group. The results are shown in the figures.

### Filtering statistics and classification

**Fig. 2:**
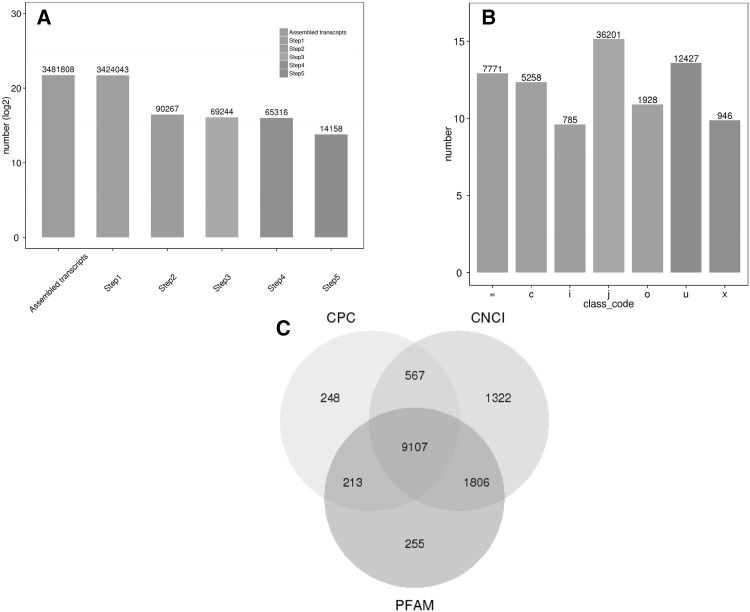
Filtering statistics, classification and coding potential screening of the lncRNAs. **a** Filtering process. **b** The classifications are shown as seven types for a complete match of the intron chain; **c** for contained; i for a trans frag falling entirely within a reference intron; j for a potentially novel isoform; o for generic exonic overlap with a reference transcript; u for unknown, intergenic transcript; x for exonic overlap with the reference on the opposite strand. **c** The coding potential was analyzed using three methods

In this study, Cufflinks was used to splice the lncRNAs. A total of 14,158 lncRNAs were selected after the five-step filtering process (Fig. 2a), including 12,427 lincRNAs, 785 intronic lncRNAs and 946 anti-sense lncRNAs (Fig. 2b). The key criterion for whether a transcript was deemed a lncRNA was whether it had coding potential. To reduce the false positive rate, the final set of lncRNAs represented the intersection of the lncRNA sets from three commonly used analysis methods: CPC, CNCI and Pfam-protein domain analysis. Figure 2c shows the number of noncoding transcripts for each method and the number (9107) of common and unique transcripts among the three methods.

### Genomic features of the lncRNAs

**Fig. 3:**
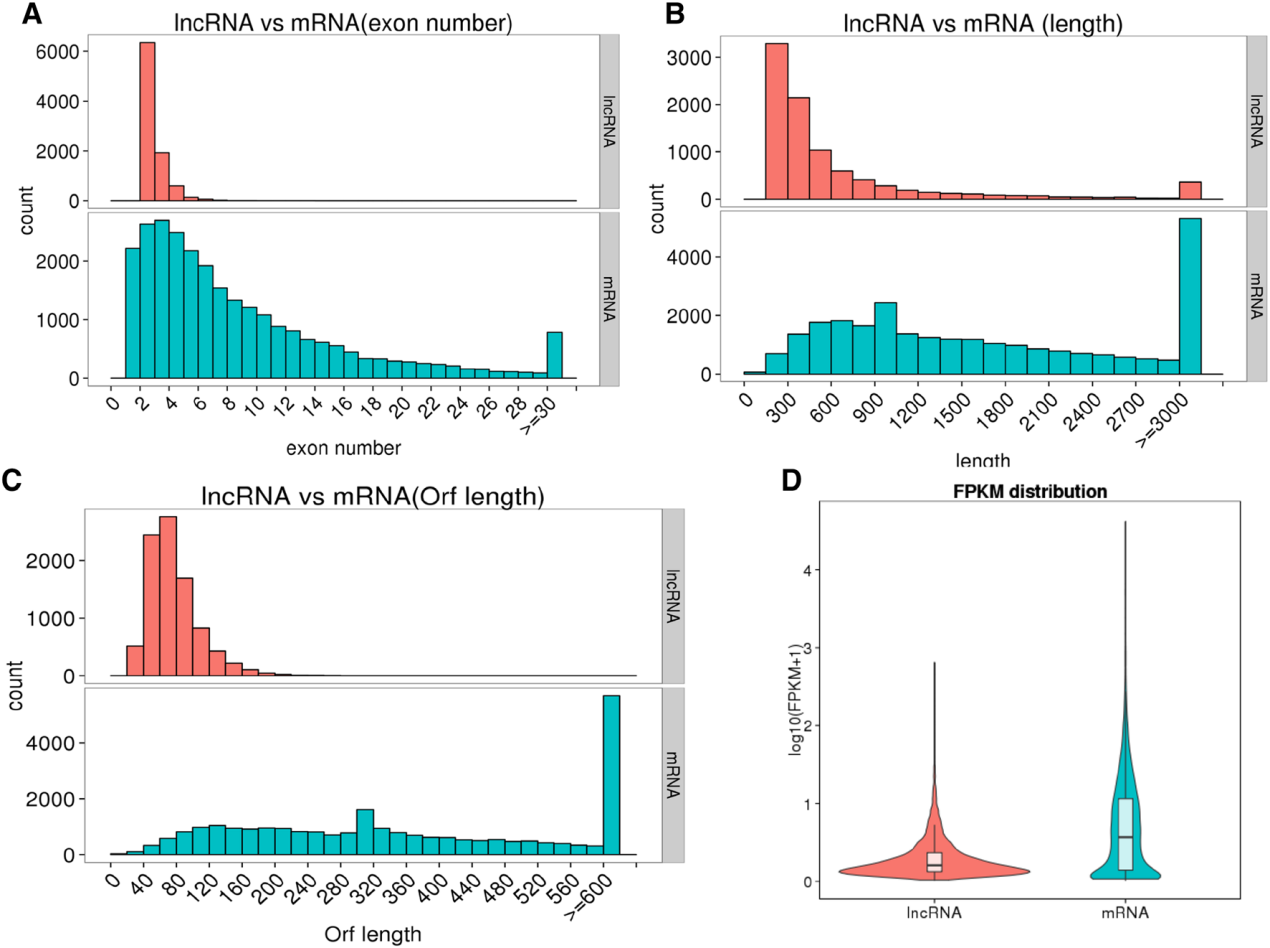
Structural comparison between lncRNAs and mRNAs. The lncRNA and mRNA transcripts were compared by exon number (**a**), length (**b**), ORF length (**c**) and FPKM value (**d**)

All of the lncRNA sequences in LW and DB pigs were assembled, and they are reported for the first time in this study. The lengths of the lncRNAs were between 200 nt and 800 nt; in contrast, the lengths of the mRNAs were more than 3000nt (Fig. 3a). Most of the lncRNAs contained 2 exons, and most of the mRNAs had approximately 3–4 exons, with fewer transcripts being observed with more exons (Fig. 3b). The ORFs of the mRNAs were acquired using structural annotation based on known genes, and the sequences were predicted using ESTScan. The ORF sequences were translated to protein sequences. The length of the predicted ORFs in the lncRNAs was centered at approximately 80nt, and this number decreased with increasing ORF length. In contrast, the lengths of most of the mRNA ORFs were longer than 600nt (Fig. 3c). The difference in the expression levels of the lncRNAs and mRNAs is expressed as the log 10 (FPKM + 1) value of the average of the two groups, and Fig. 3d shows the average expression levels for the lncRNAs and mRNAs.

### Characteristics of the expression levels of the lncRNAs and mRNAs

**Fig. 4:**
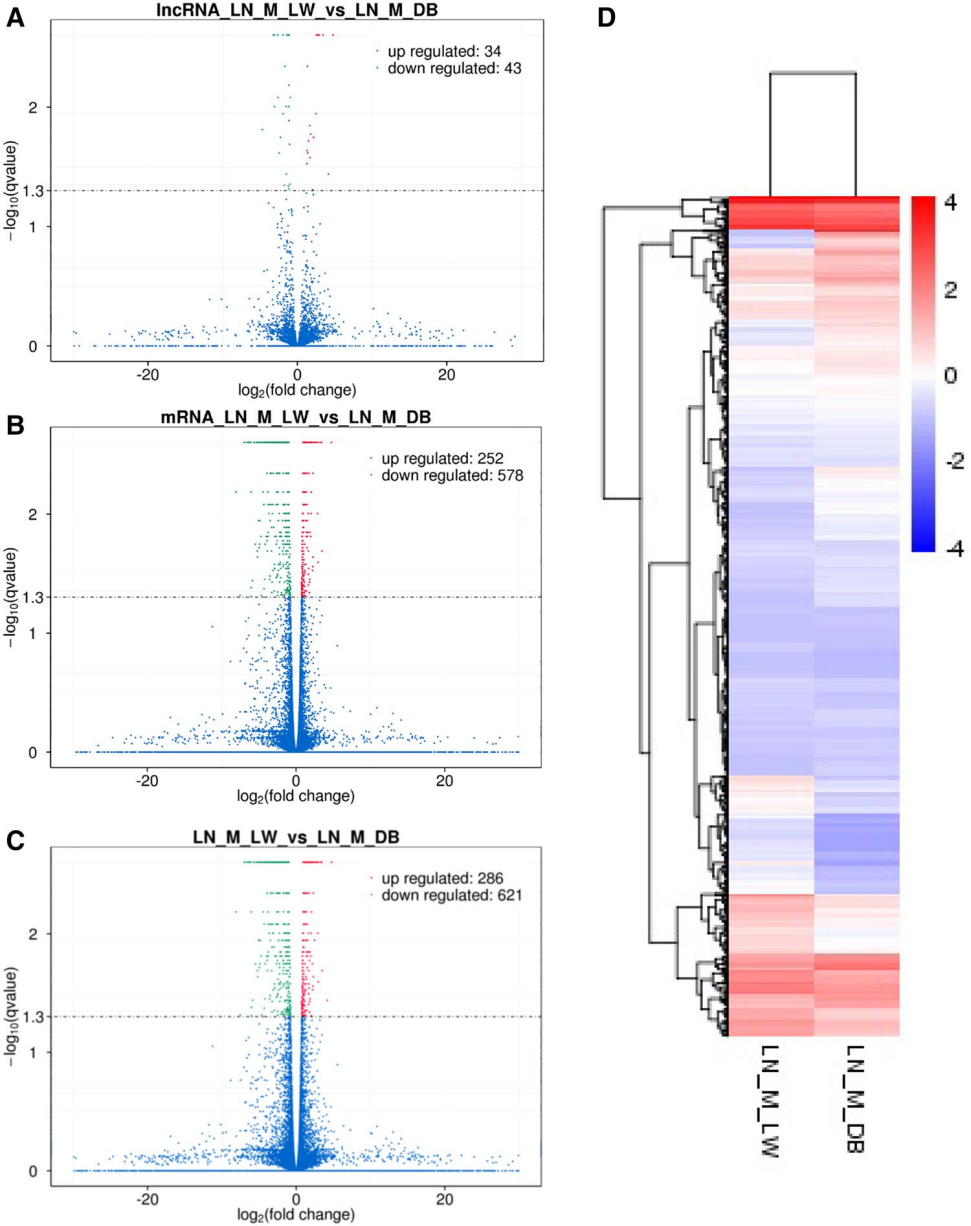
Characteristics of the lncRNA expression levels. **a** Differentially expressed lncRNAs; red: upregulated, green: downregulated and others: blue. **b** mRNA expression levels. **c** The expression levels of all the lncRNAs and mRNAs, including those that were upregulated (286) and downregulated (621). **d** The heat map shows the expressed lncRNAs (P < 0.05) in the two groups. (Color figure online)

**Fig. 5:**
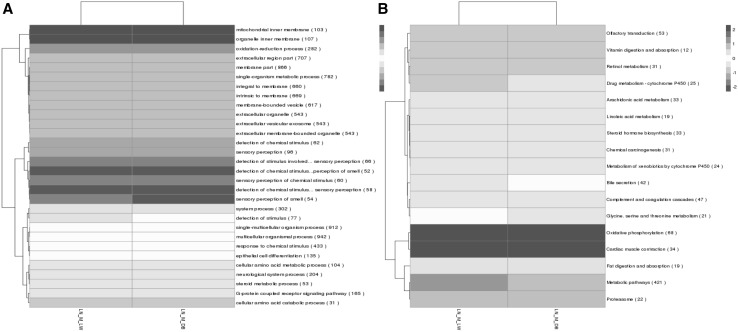
Heat map of the GO terms (left panel, **a**) and KEGG terms (right panel, **b**) for the trans lncRNAs

The selected lncRNAs were quantified using Cuffdiff software for the read count and FPKM analyses. The graphs in Fig. 4a, b show the lncRNA and mRNA expression levels. There were 907 differentially expressed lncRNAs (P < 0.05), 286 of which were upregulated and 621 were downregulated (Fig. 4c). 286 lncRNAs were expressed higher in lean pigs than fatty pigs, and 621 were expressed lower in lean pigs than fatty pigs. The heat map (Fig. 4d) shows the differentially expressed lncRNAs and mRNAs (P < 0.05) between the LW and DB groups. The expression patterns were clearly different between the two groups (Fig. 5).

### GO and pathway analyses for the intersection genes

**Fig. 6:**
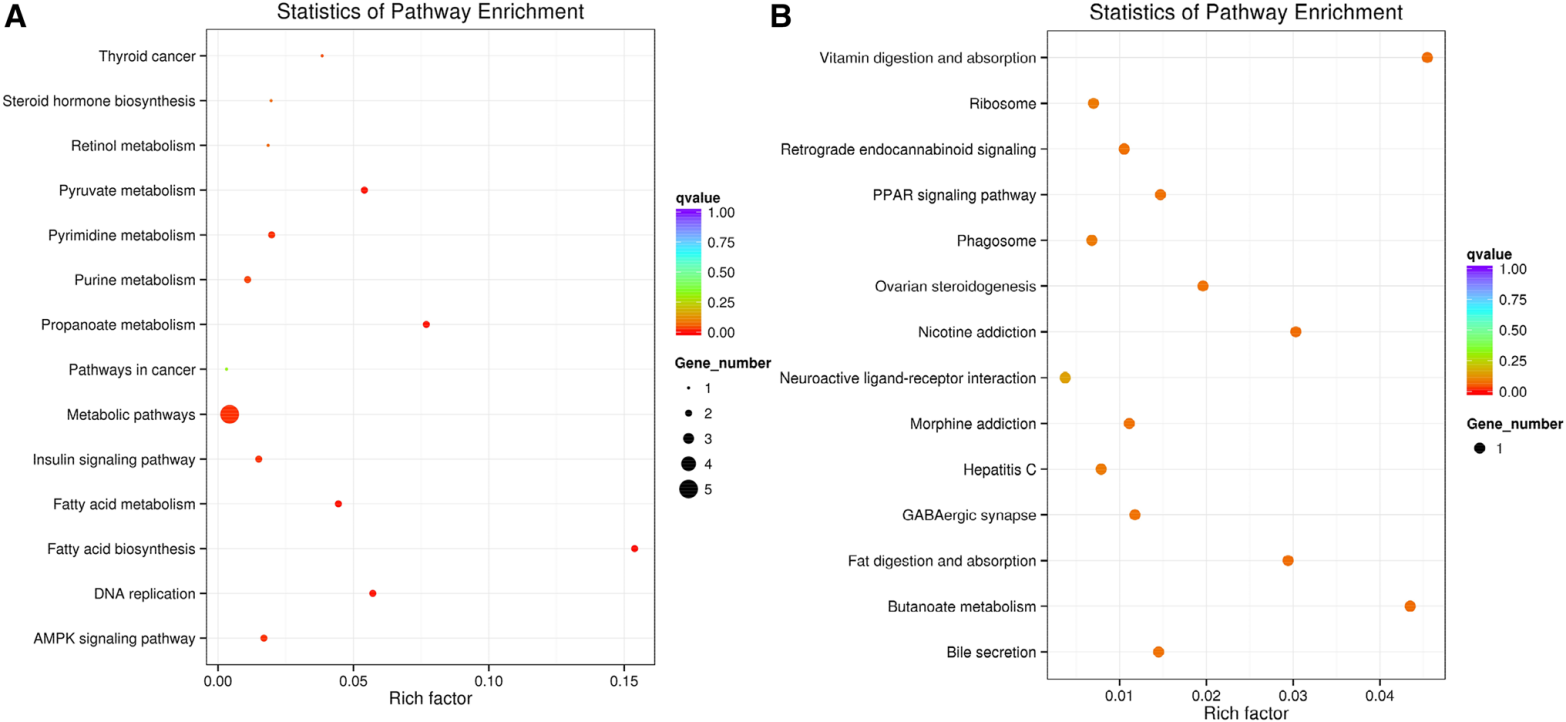
Cis lncRNA GO terms and KEGG pathways. **a** GO classification (downregulated) analysis showed enrichment mainly for AMPK signaling pathway, fatty acid biosynthesis, fatty acid metabolism and insulin signaling pathway. **b** KEGG pathway analysis for the downregulated and upregulated pathways, which were mainly enriched for fat digestion and absorption and the PPAR signaling pathway

The criterion for identifying cis target genes was that the lncRNAs should be in relatively close proximity to the protein-coding genes (Ørom et al. 2010). Therefore, all genes in the proximity of the lncRNA loci (10 kb or 100 kb upstream or downstream) were selected as target genes, and the enrichment of specific molecular functions among the target genes was analyzed to predict the functions of the lncRNAs. In GO classification, LN_M_LW and LN_M_DB pigs were mainly enriched for the GO classifications biological process and molecular function. In contrast, the KEGG pathway analysis revealed that the main downregulated pathways included metabolic pathways, the AMPK signaling pathway, fatty acid biosynthesis, fatty acid metabolism and the insulin signaling pathway (Fig. 6a). One target gene was upregulated in fat digestion and absorption (Fig. 6b). This provides further evidence that many lncRNAs may be involved in fat deposition in LN_M_LW and LN_M_DB pigs. Therefore, future studies are needed to validate these predictions and determine the role of these lncRNAs in fat deposition.

**Fig. 7:**
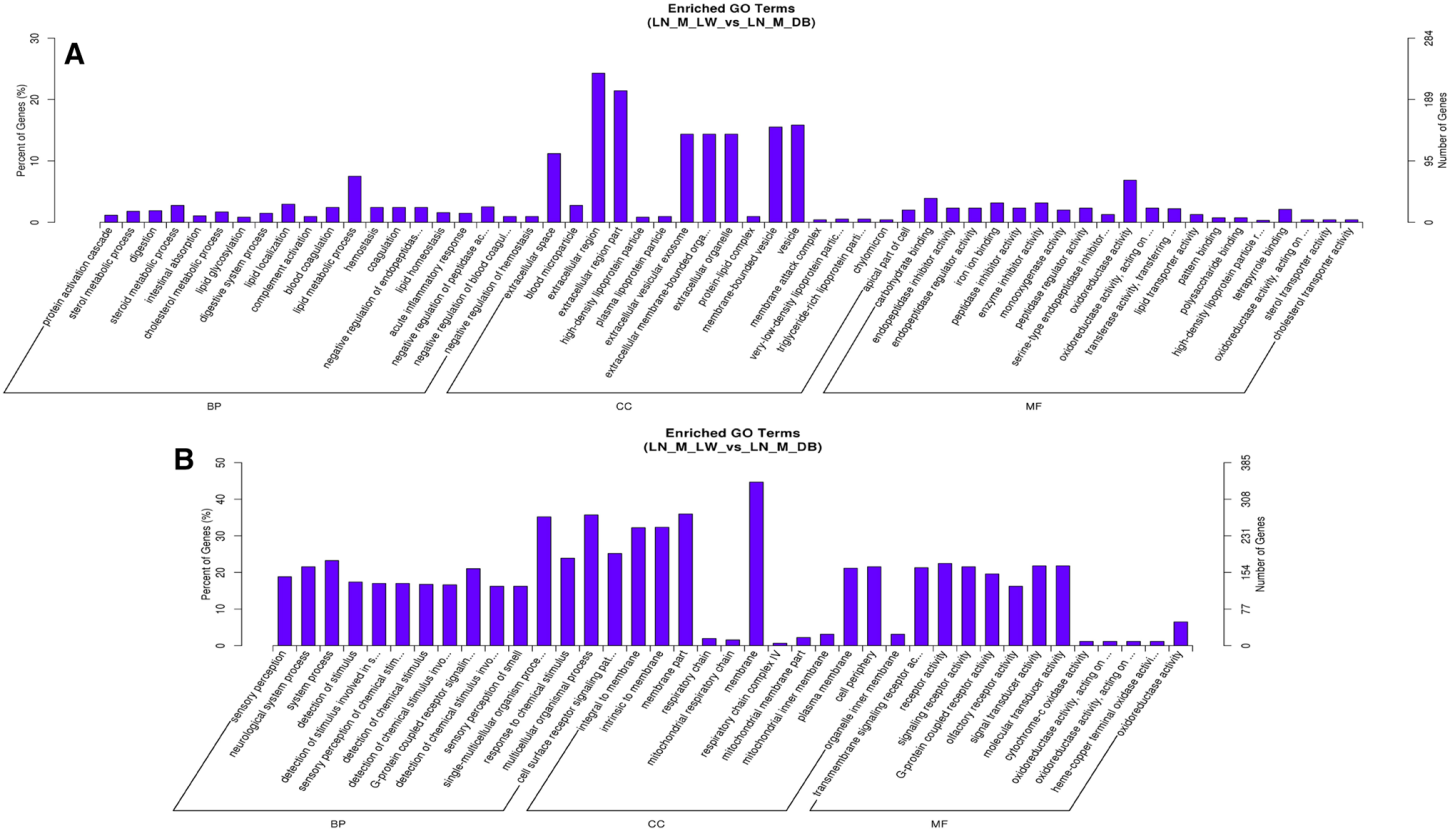
Trans lncRNA GO terms and KEGG pathways. **a** GO classification (downregulated) analysis of the trans lncRNAs showed enrichment for extracellular region, extracellular region part, membrane-bounded vesicle and vesicle. **b** The upregulated trans lncRNAs were enriched for adipocytokine signaling pathway and fatty acid metabolism

The trans target gene predictions were based on correlations between the lncRNA and mRNA expression levels (Richards et al. 2015). The main biochemical metabolic pathways and signal transduction pathways of the differentially expressed lncRNA-regulated target genes were determined by pathway enrichment (Xia et al. 2016). A GO enrichment histogram intuitively shows the number of genes (downregulated) for a term distributed across the GO classifications biological process, cellular component and molecular function. The results showed enrichment for several GO terms, including extracellular region, extracellular region part, membrane-bounded vesicle and vesicle (Fig. 7a). These enriched pathways were primarily related to retinol metabolism, complement and coagulation cascades, bile secretion, steroid hormone biosynthesis and chemical carcinogenesis.

The upregulated GO terms for the trans target genes are shown in Fig. 7b. The results showed that the GO terms were also distributed across biological process (bp), cellular component (cc) and molecular function (mf). Enrichment was seen for the GO terms membrane, membrane part, multicellular organismal process, single-multicellular organism process, intrinsic to membrane and integral to membrane. KEGG pathway analysis (up-regulated) for the two types of pigs indicates that many of these pathways were related to fat deposition such as nonalcoholic fatty liver disease (NAFLD), the adipocytokine signaling pathway and fatty acid metabolism. Some disease-associated pathways were enriched, including Alzheimer’s disease, oxidative phosphorylation, Parkinson’s disease and Huntington’s disease. Thus, these results open up new ideas for studying human obesity and disease.

### Validation of the lncRNAs

**Fig. 8:**
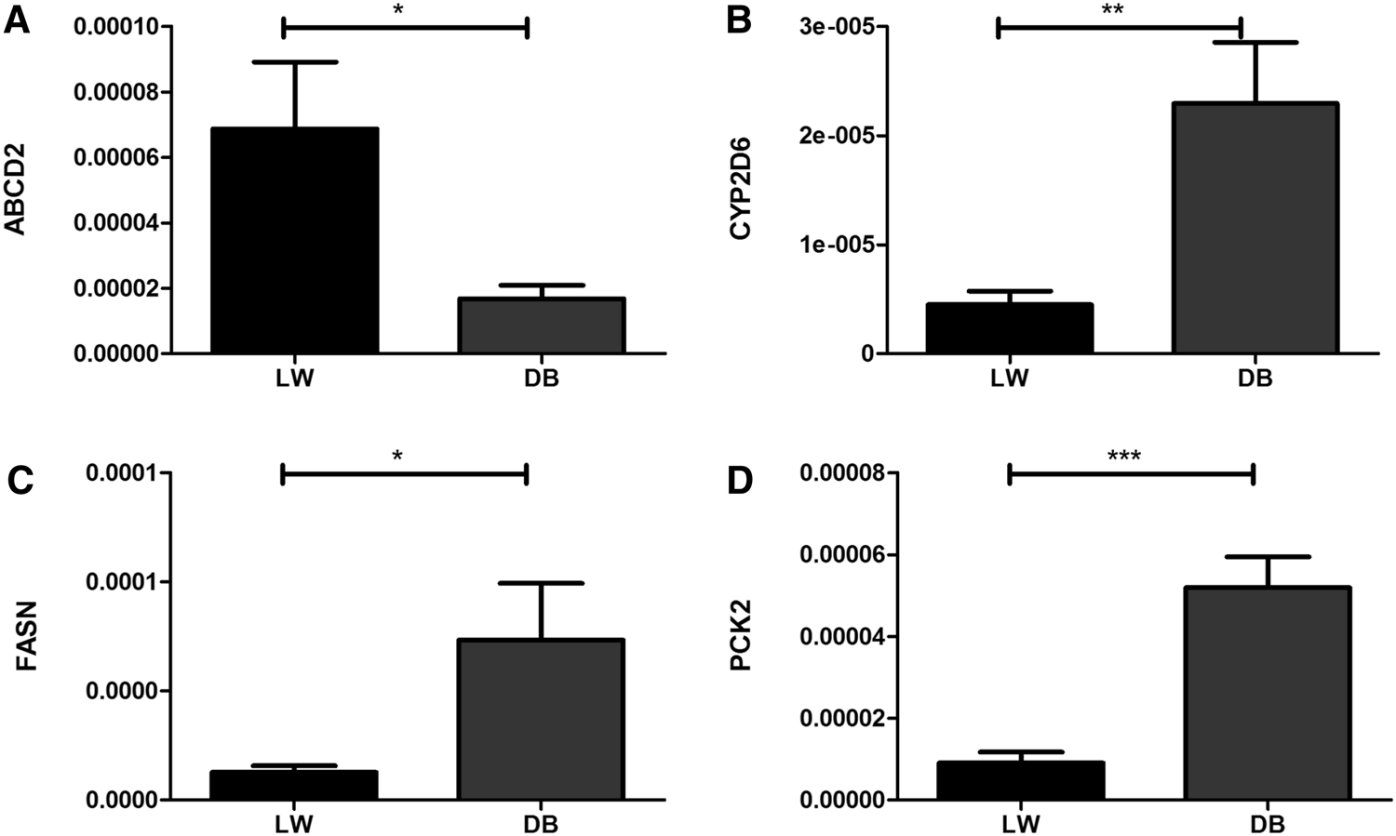
Validation of several lncRNAs. **a** ABCD2, **b** CYP2D6, **c** FASN, **d** PCK2

This study identified many lncRNAs that may be involved in fat deposition in LN_M_LW and LN_M_DB pigs. Several lncRNAs haven been validated (partially listing in Fig. 8). However, future studies will be needed to validate these predictions and determine the role of these lncRNAs in fat deposition. Interestingly, based on our previous studies, the lncRNAs ADD1 (geneid: ENSSSCG00000008692, geneid: ENSSSCG00000023124) and DGAT1 (geneid: ENSSSCG00000005918) have been confirmed to play important roles in the process of fat deposition. In LW pigs, ADD1 mRNA was expressed at the highest level in adipose tissue, and its expression was significantly different between fat and muscle tissues (P < 0.05). ADD1 mRNA expression in muscle was positively correlated with the IMF content (P < 0.05) (Cui et al. 2015). The expression of the DGAT1 gene was positively correlated with BFT, suggesting that the mechanism of IMF and BFT is different (Cui et al. 2011).

## Discussion

Recent studies have revealed that many lncRNAs play pivotal roles in regulating adipocyte development, gene expression and biological processes (Wei et al. 2016a, b; Sun et al. 2015). Thus, lncRNAs could represent a new approach for designing therapeutic and diagnostic methods (Stelzer et al. 2014). Many studies have focused on the differences between different time points in the same tissues (Ran et al. 2016) or different developmental phases (Zhao et al. 2015; Xia et al. 2016), whereas the differences in lncRNAs in the longissimusdorsi muscle of different porcine breeds have not been fully illustrated. Furthermore, the mechanisms by which lncRNAs affect fat deposition remain unclear. Pigs have become a widely used experimental animal model in human disease research, particularly for treatment strategies and the development of novel drugs, due to their similarities with humans (Ulitsky and Bartel 2013). Therefore, in this study, we identified for the first time 907 lncRNA transcripts from the longissimusdorsi muscles of LN_M_LW(fatty) and LN_M_DB(lean) pigs.

In this study, various differentially expressed lncRNAs were identified that appear to regulate target protein-coding genes that are closely associated with fat deposition. The lncRNAs identified from LN_M_LW(fatty) and LN_M_DB(lean)pig muscles share some specific characteristics with those of mice, rats, humans and other mammals, such as fewer exons, shorter exon lengths and lower expression levels than protein-coding genes (Derrien et al. 2012; AOAC 2000; Ravasi et al. 2006; Cabili et al. 2013; Billerey et al. 2014). These clues further indicated that mammals have many similar characteristics and provide a reference for studying lncRNAs in other species.

### LncRNAs regulate target genes

LncRNAs can regulate target genes in cis or trans, both of which could be important for the regulation of gene expression, transcription and posttranscriptional modification. A functional enrichment analysis of differentially expressed lncRNA target genes was carried out to search for relevant GO terms and pathways associated with cis and trans regulation in LW and DB pigs.

Bioinformatic analysis suggested that some lncRNAs are involved in important biological processes associated with fat deposition, such as the adipocytokine signaling pathway, fatty acid metabolism, the AMPK signaling pathway and fat digestion and absorption. Importantly, we found many pathways involved in obesity-related diseases, such as Alzheimer’s disease, Parkinson’s disease and NAFLD, and in fat deposition in muscle, such as the insulin signaling pathway. Here, we validated an important pathway gene (PPARγ) related to fat deposition. PPARγ has important roles in controlling the IMF content and adipocyte differentiation in pigs (Cui et al. 2016). PPARγ also regulates adipogenesis, whole-body insulin sensitivity and lipogenesis (Marion-Letellier et al. 2016). Therefore, further studies of the molecular mechanisms associated with intramuscular fat deposition in the PPAR signaling pathway could provide new information for the treatment of obesity and obesity-related diseases in humans. Although the role of lncRNAs in pigs has not yet been fully elucidated, this study serves as a resource on lncRNAs to further understand their roles in the regulation of fat deposition and obesity in humans.

In conclusion, this is the first report on the lncRNAs in the longissimusdorsi muscle of fatty- and lean-type pigs based on a RNA-seq approach. We identified two lncRNAs that are closely related to fat deposition according to their target genes. Finally, we identified the function of these lncRNAs to clarify the molecular mechanisms of fat deposition in pigs, which may serve as a reference for studies of human obesity.

## Materials and methods

### Ethics statement

This experiment was performed in accordance with the Institutional Animal Care and Use Ethics Committee of Shandong Agricultural University (No. SDAUA-2011-013) and the “Guidelines for Experimental Animals” of the Ministry of Science and Technology (Beijing, PR China).

### Collection and detection of the samples

The experimental cohort consisted of 3 LW and 3 DB pigs. This sample size is acceptable in pig physiology research (Yang et al. 2017). All pigs were housed at the Laiwu Breeder Pig Farm Co., Ltd. (Laiwu city, Shandong Province, China) and fed a diet formulated to meet current nutritional requirements. On the day of slaughter, the mean weight of the pigs was 114 ± 2 kg. Samples were collected from the longissimusdorsi muscle at the last rib and a portion of the muscle tissue. IMF was chemically quantified following ISO 1443:1973. The method used was direct Soxhlet extraction of fat by a solvent (Langmead et al. 2009). To measure the BFT, the thickness of the subcutaneous fat between the sixth and seventh thoracic vertebrae was measured with Verniercalipers. Afterward, the remaining samples were stored at − 80 °C for further analysis.

### The fatty and lean library preparation and sequencing

The fatty and lean library preparation and sequencing were performed by Novogene Bioinformatics Technology Corporation. For the specific experimental steps, see the Supplementary documentation. Briefly, total RNA was isolated from 3 LW pigs (fatty) (numbered LN_M_LW1, LN_M_LW2, and LN_M_LW3) and 3 DB pigs (lean) (numbered LN_M_DB1, LN_M_DB2, and LN_M_DB3). The ribosomal RNA (rRNA) was removed using an rRNA removal kit (Epicentre, WI, USA), and then, the sequencing libraries were generated. cDNA fragments between 150 and 200 bp were selected and purified with an appropriate system (Beckman Coulter, Beverly, USA).

Subsequently, the libraries were sequenced on an Illumina HiSeq4000 platform, and 500-bp paired-end reads were generated according to the manufacturer’s instructions. The sequencing read type was PE150 for the HiSeq4000 platform. This strategy allows paired-end 150-bp standard reads. The fragment insert size for the library construction was 250–300 bp.

### Transcriptome assembly

Clean data were obtained from the raw data after 5 steps to filter out reads with adaptors and poly-N values > 10% and low-quality reads through the use of in-house Perl scripts developed by the Novogene Bioinformatics Institute (Beijing, China). The 5′ adapter used in this study for sequencing is 5′-AATGATACGGCGACCACCGAGATCTACACTCTTTCCCTACACGACGCTCTTCCGATCT-3′ (part #15013205). The index of the pig reference genome was constructed using Bowtie (Guttman et al. 2010), and the paired-end clean reads were aligned using TopHat (Trapnell et al. 2009). The mapped reads of each sample were assembled by Cufflinks.

### Validation of differentially expressed lncRNAs

SYBR green real-time PCR amplification was conducted. The 2-ΔΔCt(ΔΔCt = ΔCt of the target gene-ΔCt of the housekeeping gene) method was used to analyze the relative quantitative data (Livak and Schmittgen 2001; Yuan et al. 2008).

### Statistical analysis

The data were analyzed on the SAS software platform (The SAS Institute, Cary, N.C., USA). The results in this article are presented as the means ± standard error of the mean (s.e.m.) (Erkens et al. 2006). Statistically significant and extremely significant differences were set at P < 0.05 and P < 0.01, respectively.
